# Effects of nursing support workers participation on negative emotions, quality of life and life satisfaction of patients with cerebral hemorrhage: a quasi-experimental study

**DOI:** 10.1186/s12912-022-01040-8

**Published:** 2022-09-19

**Authors:** Qinglian Luo, Xingzhao Luan, Chengling Xia, Liming Hou, Qisheng Wang, Mingkuan Zhao, Hua Tang, Haowen Zheng, Haiping He, Wei Xiang, Jianmei Wang, Jie Zhou

**Affiliations:** 1 Department of Neurosurgery, The Affiliated Hospital of Southwest Medical University, Southwest Medical University, Taiping Street 25#, Luzhou, 646000 Sichuan Province China; 2 Sichuan Clinical Research Center for Neurosurgery, Luzhou, 646000 China; 3 Academician (Expert) Workstation of Sichuan Province, Luzhou, 646000 China; 4 Southwest Medical University, Luzhou, 646000 China; 5 Department of Neurosurgery, Hejiang County People’s Hospital, Sichuan Province, Luzhou, 646000 China; 6 Department of Pathology, Affiliated Hospital of Southwest Medical University, Taiping Street 25#, Luzhou, 646000 Sichuan Province China

**Keywords:** Cerebral hemorrhage, Nursing, Nursing support workers, Negative emotions, Quality of life

## Abstract

**Background:**

Due to the high nursing pressure of patients with cerebral hemorrhage and the general shortage of clinical nurses, nursing support workers often participate in clinical nursing work, but the influence of nursing support workers' participation on the negative emotion, quality of life and life satisfaction of patients with intracerebral hemorrhage is unknown.

**Methods:**

This quasi-experimental study was conducted with a pretest–posttest design. A total of 181 ICH patients admitted to our hospital from January 2022 to April 2022 were enrolled, including 81 patients receiving conventional care (CG control group) and 80 patients receiving nursing support worker participation (RG research group). All patients were recorded with self-perceived Burden Scale (SPBS), Hamilton Depression Scale (HAMD), Quality of Life Scale (SF-36), Somatic Self rating Scale (SSS), Patient self-care ability assessment scale (Barthel) and Satisfaction with life scale (SWLS) scores.

**Results:**

Patients with high negative emotion were more willing to participate in clinical nursing work (*p* < *0.05*). Nursing support workers involved in cerebral hemorrhage patients can alleviate negative emotions, improve life quality, improve life satisfaction (*p* < *0.05*).

**Conclusion:**

The participation of nursing support workers can alleviate the negative emotions of ICH patients, enhance their self-management ability, and improve their life quality.

**Supplementary Information:**

The online version contains supplementary material available at 10.1186/s12912-022-01040-8.

## Background

Intracerebral hemorrhage (ICH) has the highest mortality of all stroke diseases, accounting for about 28% of all stroke patients [1–4]. In recent years, the incidence of intracerebral hemorrhage tends to be younger, and the incidence of the young and middle-aged people around 40 years old shows an increasing trend [3, 5]. Cerebral hemorrhage has a rapid onset and development. While causing limb dysfunction, negative emotions are also accompanied by disease and exist for a long time [1, 4, 6]. Intracerebral hemorrhage in the face of disease will produce anxiety, depression and other adverse emotions, not only affect the effect of surgical treatment, even affect postoperative rehabilitation, increase the incidence of complications [7]. However, medical nurses registered in the world are generally facing the problem of staff shortage and heavy workload, which also leads to cerebral hemorrhage patients are prone to complications, especially psychological problems are easier to be ignored [8, 9]. Therefore, correct nursing and timely intervention of negative emotions during hospitalization are very important.

The increase in the incidence of diseases such as cerebral hemorrhage over the past decade has led to an increase in international demand for nurses, often without a corresponding increase in supply [10]. This, coupled with persistent retention problems, has led to challenges in staffing hospitals in the right numbers and mix of staff [11, 12]. The emergence of care support workers in the face of increased nursing workloads and projected labor shortages has helped alleviate some of these problems [11]. Increased care support staff was found to increase patient contact (providing more hours of care) and to improve the quality of care and reduce the risk of death for patients compared with a control group matched by workload classification [13, 14]. However, whether nursing support workers can affect negative emotions, quality of life and life satisfaction of patients with clinical intracerebral hemorrhage remains unclear.

## Methods

### Design

This quasi-experimental study was conducted using a pretest–posttest design. The objective is to investigate the effect of nursing support workers on negative emotions, quality of life and life satisfaction of patients with cerebral hemorrhage in clinical nursing.

### Participants

We recruited patients treated with cerebral hemorrhage in the neurosurgery department of Affiliated Hospital of Southwest Medical University (grade III hospital of Sichuan Province) from January 2022 to April 2022. Participants were fully informed about the purpose of the study before written consent was obtained. According to whether nursing support workers participated in clinical nursing work, the participants were non-randomly divided into the control group and the research group, including 81 people in the control group and 80 people in the research group. Patients were evaluated at admission and one month after discharge. Both groups were followed up simultaneously. Ethical Message: REDACTED.

### Inclusion and exclusion criteria

Inclusion criteria were: patients diagnosed with intracerebral hemorrhage based on medical history and imaging results, conscious, voluntary acceptance of the investigation, complete clinical data, and informed consent signed by patients and their families.

Exclusion criteria were: patients with infectious diseases or severe organ dysfunction, patients with poor compliance during treatment, patients who were transferred to hospitals or dropped out of the study, patients with physical disabilities or complicated malignant tumors.

### Distinction between nursing staff and nursing support workers

Nursing staff are staff who have obtained professional qualifications and are employed by hospitals. Nursing support workers receive only a few weeks of theory knowledge training began to practice, the role of nursing support workers main is making the bed, assist to eat and shower, communicate with patients, for simple bandage and transporting patients, timely report the patient's requirements and emergency to have qualification of nursing staff.

### Nursing methods

Control group: From admission to discharge, the following nursing work shall be attended by qualified nursing personnel.

1. The patient's condition was monitored, and any abnormality was reported to the doctor in time, and the attending doctor intervened.
2. Strengthen patient management, prevent falls, bedsores and other unexpected risk events.
3. Guide patients on psychological diet and living habits, and communicate with them.

Research group: Qualified nursing staff and nursing support staff participate in the following routine nursing work between admission and discharge.

1. In addition to nursing staff, nursing workers shall monitor the patient's condition together and report any abnormality to doctors in time.
2. Nursing workers participate in patient management, such as prevention of falls, bedsores, etc.
3. Nursing workers participate in the guidance of psychological diet and living habits of patients and communicate with them.

### Scoring criteria

The following scales were collected within 24 h of admission and one month after discharge.

#### Self-perceived burden scale (SPBS)

Self-perceived burden scale (SPBS) was used to evaluate the degree of self-perceived burden of college students [15]. The scale measures the burden of patients from three dimensions: physical burden (3 items), economic burden (2 items) and mental burden (5 items), with a total of 10 items. Each item is graded on a 5-point scale. The higher the score, the greater the perceived burden.

#### Hamilton Depression Scale (HAMD)

Depression was assessed with the 24-item Hamilton Depression Rating Scale (HAMD). The scale was used to assess depressive symptoms in adults in the past week, with a score of less than 8 indicating no depression, 8 indicating mild depression, 20 indicating moderate depression and more than 35 indicating major depression, the higher the score, the more severe the depression [16].

#### Hamilton Anxiety Rating Scale (HAMA)

The anxiety of the patients was assessed by the Hamilton Anxiety Scale (HAMA) [17], which is a 14-item questionnaire evaluating anxiety symptoms in the past week. It has two main components, physical anxiety and psychological anxiety. An overall score greater than 29 was considered severe anxiety; Scores of 21–29 indicated significant anxiety; A score of 14–21 indicates anxiety; A score of 7 to 14 indicates possible anxiety. A score below 7 indicates no anxiety symptoms. In general, HAMA scores higher than 14 indicate clinically significant anxiety symptoms, subjects with higher scores indicated anxiety.

#### Quality of Life Scale (SF-36)

The SF-36 scale, developed by the American Medical Research Institute, is widely recognized and used in the world [18]. The scale has 8 dimensions to evaluate health-related quality of life (HRQOL), which belong to two categories of physical health and mental health, namely physical function, physical function, physical pain, overall health, vitality, social function, emotional function and mental health, higher scores indicate higher quality of life.

#### Somatic Self rating Scale (SSS)

The SSS score scale was used to evaluate the physical symptoms of the patients at admission and one month after discharge. There were 20 questions in this table, with a total score of 80 points, with a total score of less than 30 being basically normal, 30–39 being mild, 40–59 being moderate, and more than 59 being severe.

#### Patient self-care ability assessment scale (Barthel)

Barthel Index rating scale was used to evaluate patients' self-care ability [19]. There were 10 questions with a total score of 100. A score of more than 60 indicates a mild disability, but a basic self-care life; A score of 40 to 60 indicates moderate disability, requiring assistance; A score of 20 to 40 means severely disabled and in need of a lot of help. A score below 20 indicates total disability and dependency. The higher the score, the better the improvement.

#### Satisfaction with life scale (SWLS)

SWLS scale was used to evaluate patients' life satisfaction [20]. There were 5 questions, with a total score of 35, with a score greater than 30 indicating very satisfaction, 26–30 indicating satisfaction, 21–25 indicating a little satisfaction, 20 indicating neutral, 15–19 indicating a little dissatisfaction, 10–14 indicating dissatisfaction, and less than 10 indicating very dissatisfaction. The higher the score, the more satisfied with life.

### Statistical analysis

Statistical analysis of the collected data was performed using SPSS 20.0 (IBM Corp, Armonk, NY, USA) and visualization was performed using GraphPad 7. The KS test was used to analyze the distribution of measured data. Measurements that follow a normal distribution are expressed as mean ± standard deviation (mean ± standard deviation). The independent sample T-test was used to analyze inter-group comparisons and the paired T-test was used to analyze intra-group comparisons. Count data were expressed as ratios (%) and analyzed using chi-square test, expressed as χ^2^. *p* < 0.05 indicated that the difference was statistically significant.

### Ethical considerations

Agency approval was obtained. All potential interviewees are competent adults. Researchers have no authority in hospitals. Support the principles of the Declaration of Helsinki, in particular the confidentiality and anonymity of information.

## Results

### Basic patient data

We consecutively recruited 161 patients with intracerebral hemorrhage admitted to our hospital from January 2022 to April 2022. The basic information of these patients is shown in Table 1. Among them, 81 patients receiving routine care were assigned to control group (CG). There were 50 males and 31 females, ranging from 30 to 92 years old. The remaining 80 patients receiving comprehensive care were assigned to research group (RG), including 58 males and 22 females, aged 25–85 years. There were no statistically significant differences between the control group and the research group in gender, age, blood glucose, BMI, education level, place of residence, self-evaluation of character, exercise habit, history of hypertension, etc. (*p* > *0.05*, Table 1).

**Table 1:** Basic clinical data [n (%)]

X	level	Control.Group	*Research.Group*	*p*
n		81	80	
Gender (%)	Female	31 (38.3)	22 (27.5)	0.198
	Male	50 (61.7)	58 (72.5)	
Age (mean (SD))		64.65 (10.87)	64.16 (13.40)	0.798
Blood.sugar (mean (SD))		8.21 (7.41)	8.03 (3.37)	0.850
BMI (mean (SD))		24.91 (2.55)	25.02 (3.71)	0.832
Degree.of.education (%)	Above primary school	26 (32.1)	33 (41.2)	0.298
	Primary school	55 (67.9)	47 (58.8)	
Self.evaluation.of.personality (%)	Extraverted	35 (43.2)	31 (38.8)	0.800
	Intermedius type	20 (24.7)	23 (28.7)	
	Introverted	26 (32.1)	26 (32.5)	
Place.of.residence (%)	Rural	47 (58.0)	42 (52.5)	0.585
	Urban	34 (42.0)	38 (47.5)	
Exercise.habits (%)	No	62 (76.5)	64 (80.0)	0.733
	Yes	19 (23.5)	16 (20.0)	
History.of.hypertension (%)	No	23 (28.4)	23 (28.7)	1.000
	Yes	58 (71.6)	57 (71.2)	

*SD* stands for standard deviation. *P* value was used for comparison between the control group and the research group. Chi-square test or T test was used, *p* > *0.05* indicated no statistical significance

### Patients with high negative emotions at admission were more likely to invite nursing support workers to participate in their care

**Fig. 1:**
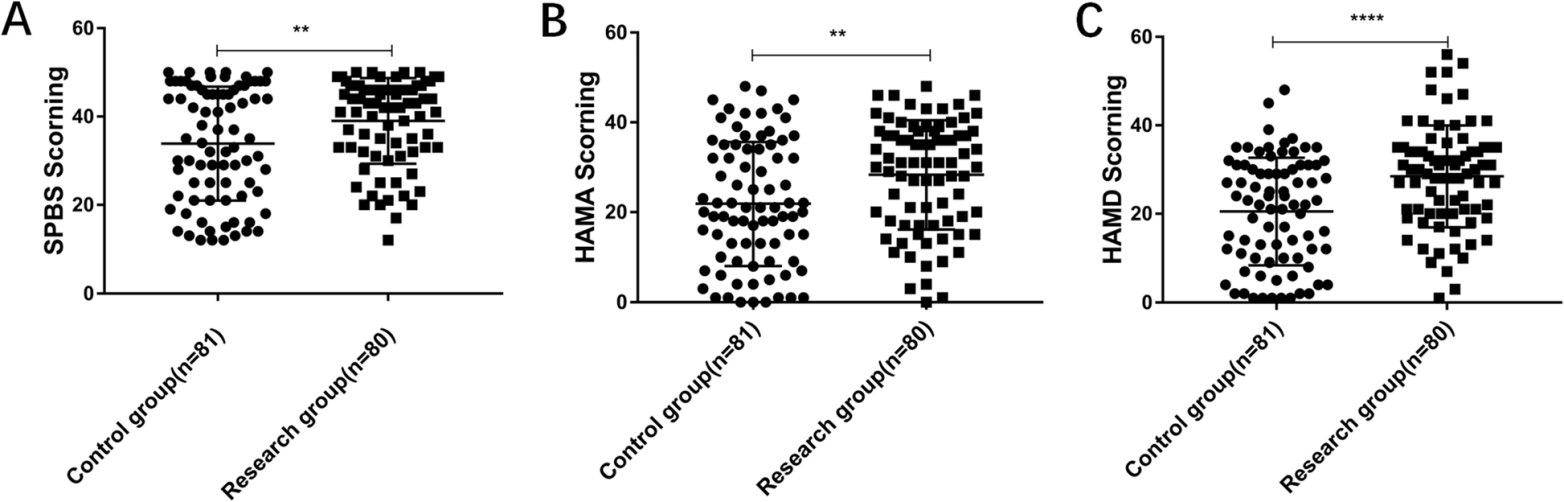
At admission, RG group had higher negative mood score than CG group. **A** The SPBS score of RG group was higher than that of CG group, **B** HAMA score in RG group was higher than that in CG group. **C** The HAMD score of RG group was higher than that of CG group (* *p* < 0.05, ** *p* < 0.01, *** *p* < 0.001)

After admission, the self-perceived burden of the research group was higher than that of the control group (*p* = *0.0048*). The anxiety degree of the research group was higher than that of the control group (*p* = *0.0020*). Similarly, the degree of depression in the research group was higher than that in the control group *(p* < *0.0001*) (Fig. 1). The results suggested that patients with intracerebral hemorrhage who had higher negative emotions after admission preferred nursing support workers to participate in clinical nursing work.

### Nursing support workers participation is beneficial to improve the negative emotions of patients with cerebral hemorrhage, improve life quality and life satisfaction

We used the score one month after discharge minus the score at admission to measure the improvement of negative emotion and quality of life and life satisfaction.

#### The participation of nursing support workers can improve the negative emotions of patients with intracerebral hemorrhage

**Fig. 2:**
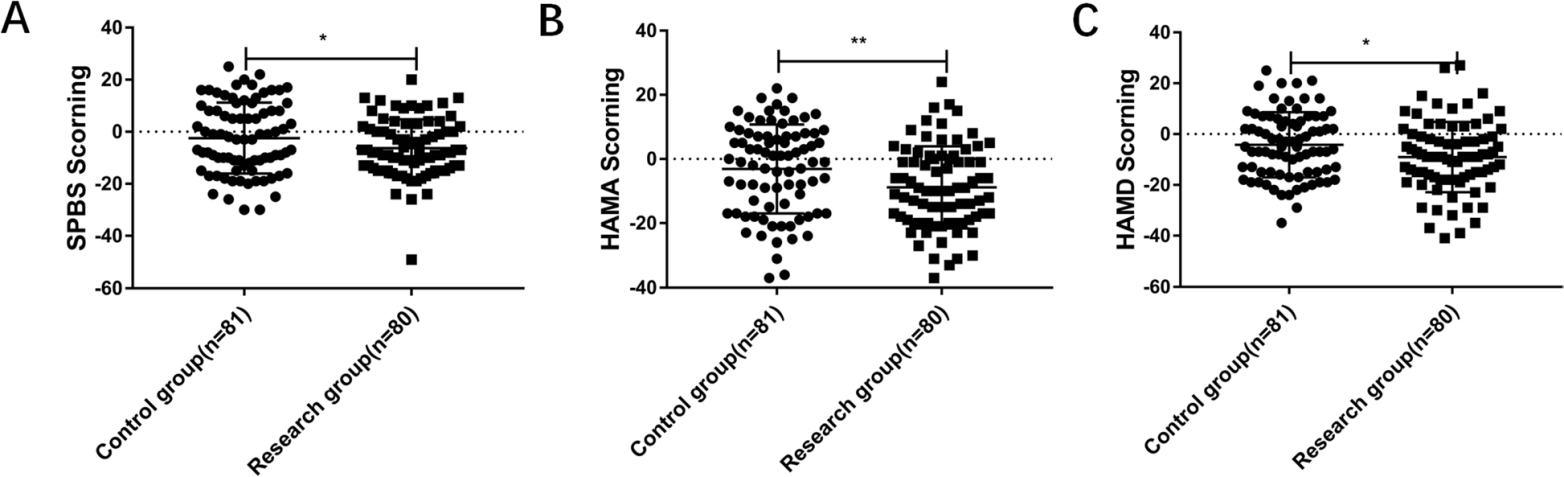
One month after discharge, RG group had better negative mood improvement than CG group. **A** SPBS score improvement one month after discharge, the lower the score, the better the improvement, **B**-**C** HAMA and HAMD scores of the two groups were compared, and the lower the score, the better the improvement degree (* *p* < 0.05, ** *p* < 0.01, *** *p* < 0.001)

We found that the difference of SPBS in the study group was smaller than that in the control group (*p* = *0.0493*). The differences in HAMA and HAMD were the same (*p* = *0.0073* and *p* = *0.0234*) (Fig. 2). These results indicate that the participation of nursing support workers in clinical nursing work is more beneficial to reduce the negative emotions of patients with cerebral hemorrhage.

#### The participation of nursing support workers is beneficial to improve the quality of life and life satisfaction of patients with cerebral hemorrhage

**Fig. 3:**
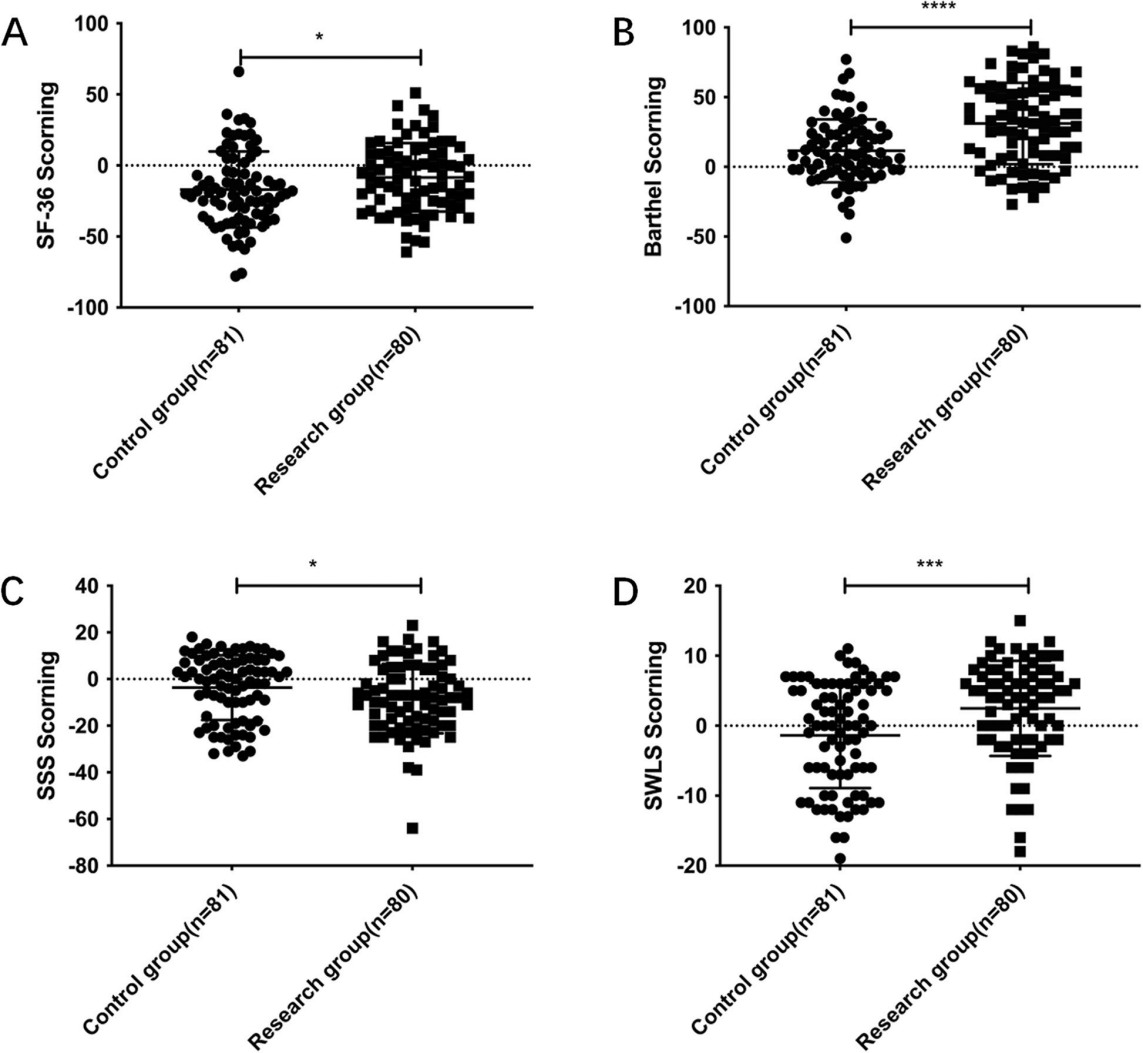
One month after discharge, the improvement of quality of life and life satisfaction in RG group was better than that in CG group. **A** Comparison of SF-36 score changes one month after discharge, **B** Barthel scores were compared between the two groups one month after discharge, **C** Comparison of SSS scores between the two groups one month after discharge, **D** SWLS scores of the two groups were compared one month after discharge (* *p* < 0.05, ** *p* < 0.01, *** *p* < 0.001)

Similarly, the score after one month minus the score at admission was used to observe the improvement of the quality of life and life satisfaction of patients. We found that SF-36 in the study group was higher than that in the control group (*p* = *0.0352*). The Barthel score was higher than that of the control group (*p* < *0.0001*). The Barthel score and the SWLS score were the same (*p* = *0.0008*) (Fig. 3). The difference of SSS in the study group was smaller than that in the control group (*p* = *0.0406*).

## Discussion

Cerebral hemorrhage is the second leading cause of death and disability in the world [21, 22]. In recent decades, with the development of medical and health care, the mortality and complications of intracerebral hemorrhage have made some progress [23]. However, nursing is still facing great pressure, pressure sores, sputum aspiration, dietary guidance and psychological counseling are still in great demand [24–26]. Therefore, it is particularly important to find a new nursing mode for patients with cerebral hemorrhage. The presence of nursing support workers can alleviate this pressure to some extent, studies have found that the allocation of nursing staff and nursing support staff in the ward will affect the patient's mortality and negative effects [14]. So, it is a positive attempt to add nursing support personnel into the nursing of patients with cerebral hemorrhage in neurosurgery.

In this study, when patients were admitted to hospital, we used SPBS, HAMA and HAMD scales to evaluate the negative emotions of patients admitted to hospital. We found that patients in the experimental group had higher negative emotion scores (*p* < 0.05). However, in our experiment, the participation of nursing support workers was chosen by patients themselves, and we did not intervene, which to a certain extent can indicate that patients with high negative emotions need more care and companionship. SPBS, HAMA and HAMD Scales were used to evaluate the improvement of patients' sexual emotions one month after discharge. The results suggested that the participation of nursing support workers could better improve the negative emotions of patients with intracerebral hemorrhage. This result indicates that the participation of nursing support workers in clinical care can better alleviate the negative emotions of patients with intracerebral hemorrhage, which is consistent with previous reports [27]. Reasons for nursing support workers to do a good job in basic nursing in patients with cerebral hemorrhage at the same time, pay attention to the establishment of a good doctor-patient relationship, patients with the understanding of basic data and negative emotional guidance, as the bridge between patients and medical staff, understand the source of the patients with negative emotions, fundamentally solve the concerns of the patients, and then improve their negative emotions.

With the continuous progress and development of clinical nursing, the ultimate goal of nursing is no longer limited to simply prolonging the survival time of patients, but more attention is paid to improving the quality of life and life satisfaction of patients. Our study suggests that the involvement of nursing support workers improved the quality of life and life satisfaction of patients. The analysis reason is that the participation of nursing support workers can improve the mental health status of patients, improve the physical, social, psychological and material life functions of patients, so as to improve the quality of life of patients. At present, there is a general shortage of clinical nurses, so that the clinical requirements and needs of patients with cerebral hemorrhage unable be fully met, and the emergence of nursing support workers is conducive to improve this situation [28]. Therefore, we believe that the participation of nursing support workers enables patients with cerebral hemorrhage to receive better clinical care, meet psychological and physical needs, and improve patients' negative emotions, quality of life, and life satisfaction. However, the study was subject to certain limitations. For example, nursing support workers' training level, proficiency level and other reasons make this study not comprehensive. We will include more types of care support workers in future studies to complement our results and make them more comprehensive.

## Conclusions

In conclusion, our study suggests that the participation of nursing support workers in clinical nursing work can better improve the negative emotions of patients with cerebral hemorrhage, improve the quality of life and life satisfaction, which is a good choice in clinical nursing work.

## Supplementary Information

**Additional file 1.**

## Data Availability

All data generated or analysed during this study are included in this published article [and its supplementary information files].
